# Carotid body paraganglioma: a case report

**DOI:** 10.11604/pamj.2023.44.182.38636

**Published:** 2023-04-19

**Authors:** Swaragandha Shivaji Jadhav, Avinash Parashuram Dhok, Kajal Ramendranath Mitra

**Affiliations:** 1 Department of Radiodiagnosis, NKP Salve Institute of Medical Sciences and Research Center, Lata Mangeshkar Hospital, Nagpur, Maharashtra 440012, India

**Keywords:** Paraganglioma, carotid body tumor, chemodectomas, neck tumor, case report

## Abstract

A highly vascular glomus tumor that develops from the paraganglion cells of the carotid body is called a carotid body tumor (CBT), also known as a chemodectoma or carotid body paraganglioma (CBP). It is situated near the carotid bifurcation, where the external and internal carotid arteries splay out characteristically. We present a case of a 30-year-old woman who had a slightly tender, slightly pulsatile, and slightly ballotable swelling over the lateral aspect of the neck on the right side. The surgical resection of the tumor was done based on the diagnosis made on clinical-radiological investigations as a carotid body tumor further confirmed by a histopathological study. We also provide a summary of the research on carotid body tumors clinical and imaging manifestations, assessment, and therapy.

## Introduction

The carotid body is located on the medial side of the carotid bifurcation bilaterally, which is the biggest group of paraganglia in the head and neck [1]. Carotid body tumors (CBTs), also known as paragangliomas or chemodectomas, are uncommon neuroendocrine neoplasms that develop in glomus cells generated from the embryonic neural crest close to the carotid bifurcation. CBTs are reported to occur 1-2 times per 100,000 people [2]. Carotid body tumors, which make up 0.6% of head and neck cancers in people, are uncommon chemical receptor tumors. Most of these tumors have no symptoms and are typically found by chance during radiological imaging examinations or by clinical examination. However, in symptomatic cases, discomfort, dysphagia, and autonomic dysfunction are the most often noted symptoms [3].

The preferred course of treatment is surgical excision. Preoperative embolization and surgical planning both benefit from preoperative angiography. Embolization encourages tumor reduction and lessens bleeding during surgery. In cases where surgery is not an option or is not necessary, radiotherapy may be explored [4]. A young primigravida woman with right-sided neck swelling who was diagnosed with CBT is used as an example.

## Patient and observation

**Patient information:** a young 30-year-old female complained of right-sided neck swelling for 4 years and intermittent pain over swelling for 4-5 months. No history of fever, cough, weight loss, or anorexia was present. No history of movement of swelling with deglutition was noted. No history of swelling on the left side of the neck, bilateral axillary region, ptosis, loss of sweating, hoarseness of voice, and syncopal attacks was present. The patient was not a known case of tuberculosis, hypertension, or diabetes mellitus.

**Figure 1:**
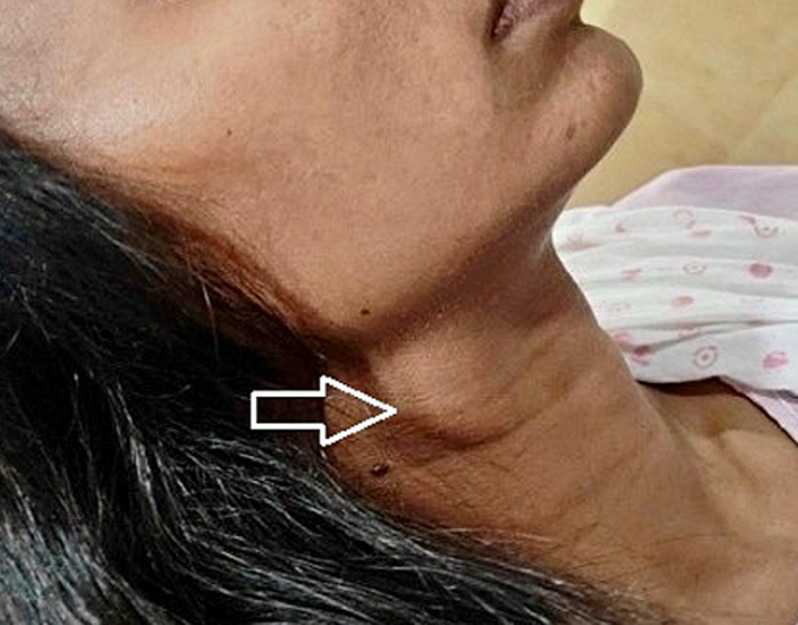
swelling below the angle of the mandible on the right side of the neck

**Clinical findings:** on local examination, a relatively well-defined swelling of approximate size 2 x 2 cm is noted at the anteromedial aspect of the sternocleidomastoid (SCM) muscle, just below the angle of the mandible on the right side of the neck (Figure 1). The swelling was firm-hard in consistency, slightly tender, slightly pulsatile, and slightly ballotable on side-to-side movement. No evidence of a local rise in temperature was noted. Bilateral carotid arteries were palpable. On general examination, she was stable and afebrile with a pulse rate of 90 beats per minute and blood pressure (BP) of 120/80 mmHg. Breast examination and systemic examination were within normal limits. Laboratory investigations such as complete blood count (CBC), thyroid function test (TFT), kidney function test (KFT), and liver function test (LFT) were within normal limits.

**Figure 2:**
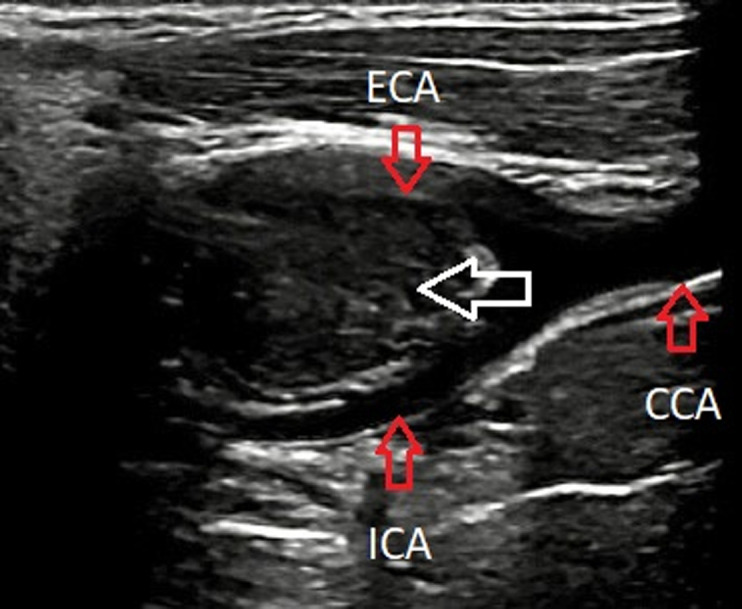
a well-defined homogenously isoechoic lesion above the bifurcation of the right common carotid artery on B mode ultrasonography

**Figure 3:**
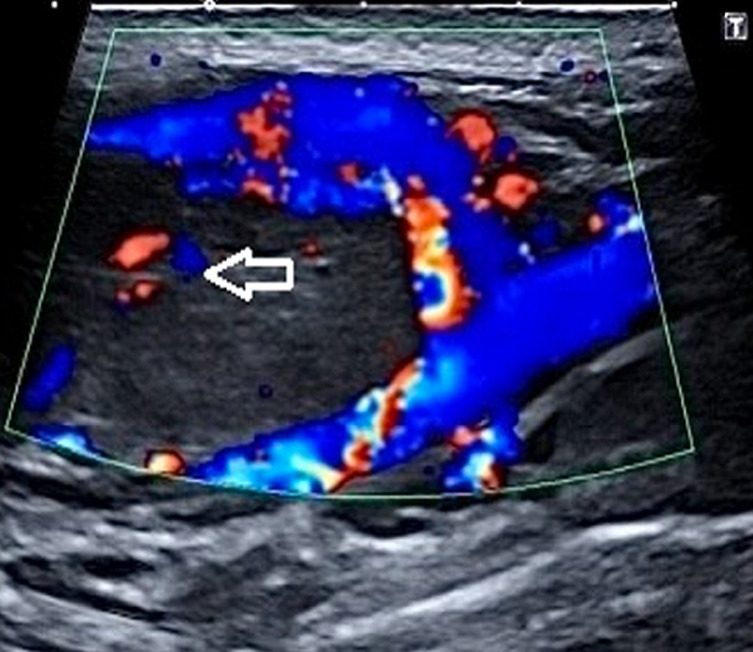
minimal vascularity within the lesion on color Doppler (arrow)

**Diagnostic assessment:** the patient has been further advised a neck ultrasound. On B mode ultrasonography neck, a well-defined ovoid homogenously isoechoic lesion of size 2.1 x 2.2 cm is noted just above the bifurcation of the right common carotid artery (CCA) causing splaying of the external carotid artery (ECA) and internal carotid artery (ICA). No evidence of calcification was noted within this lesion. No evidence of adjacent inflammatory changes is noted (Figure 2). On color Doppler, minimal vascularity is noted within this lesion (Figure 3). No evidence of lymph nodes is noted in the bilateral cervical region. Bilateral CCA´S, bilateral ICA´S, and bilateral ECA´S show normal course and caliber with normal grey scale features. Duplex Doppler study shows normal laminar flow in bilateral CCA, ECA, and ICA with normal flow velocities and waveform patterns. A diagnosis of the well-defined isoechoic lesion at the bifurcation of the right CCA is likely a CBT was made.

**Figure 4:**
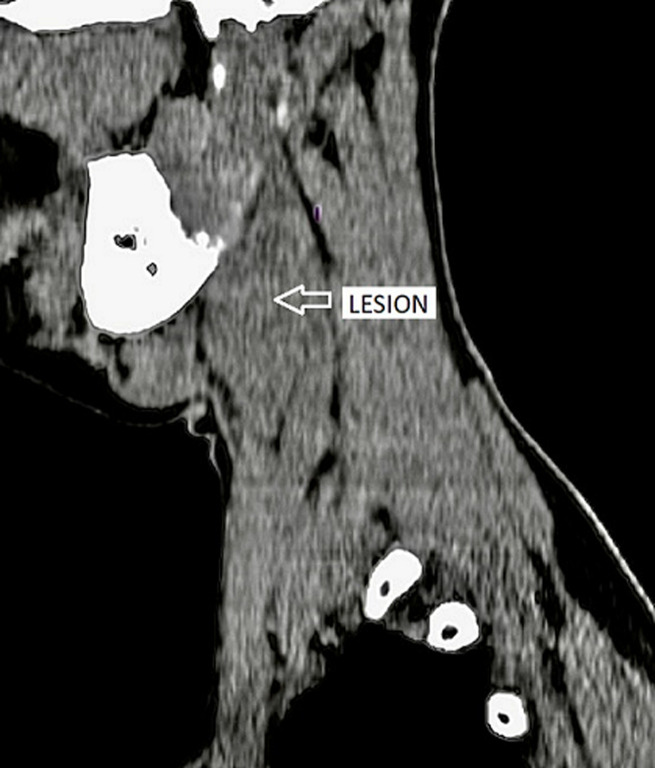
CT-scan of a bilobed soft tissue density mass lesion on the right side of the neck anterior to the sternocleidomastoid muscle (arrow)

**Figure 5:**
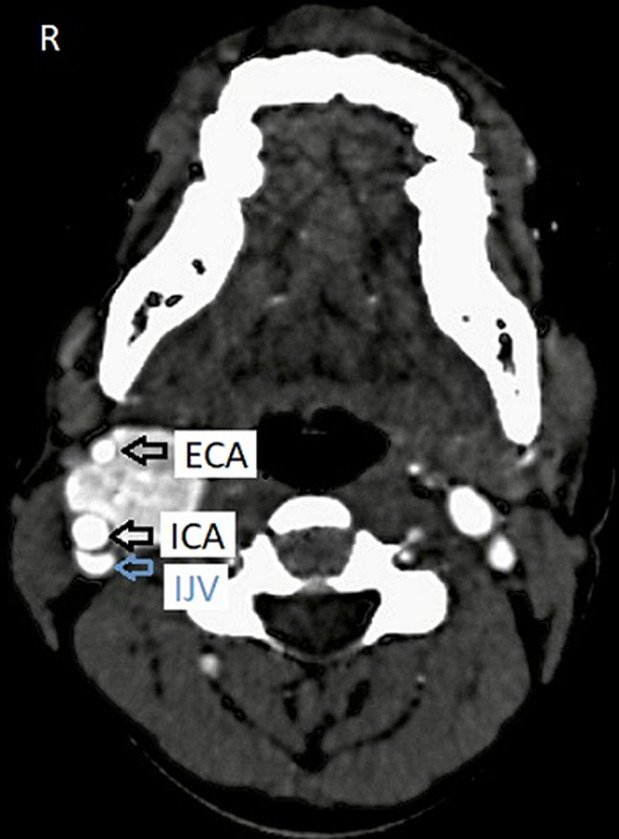
an intense homogeneous enhancement; multiple thin tortuous channels noted around this lesion after the post-contrast study axial section (arrows)

**Figure 6:**
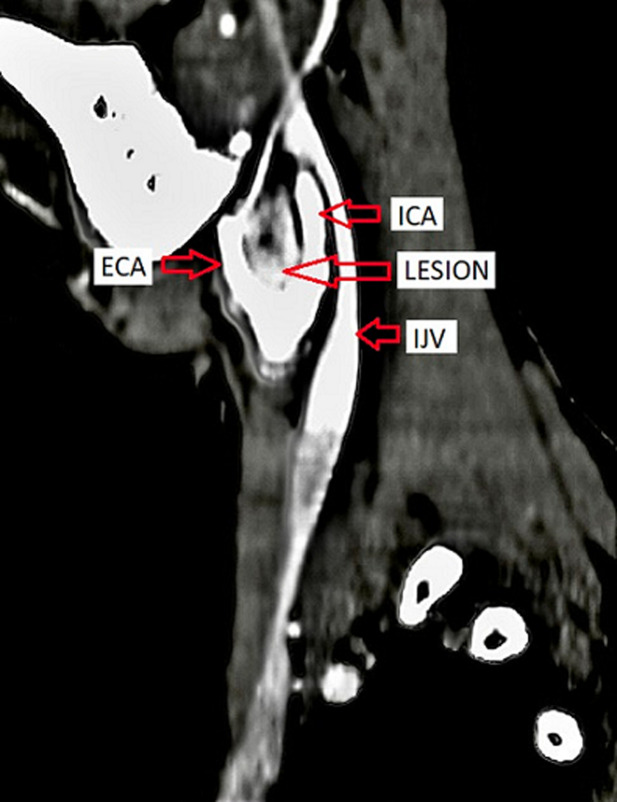
a mass lesion causing splaying of the internal carotid artery and external carotid artery on the right side after the post-contrast study sag section (arrows)

The patient was advised contrast-enhanced computed tomography (CECT) of the neck for further confirmation. On plain computed tomography (CT) sagittal (sag) section, an ill-defined soft tissue density mass lesion is noted on the right side of the neck anterior to the SCM muscle inferior to the angle of the mandible at the level of the hyoid bone extending superiorly up to suprahyoid space at C4-C6 vertebral level. No evidence of calcification was noted within (Figure 4). On the post-contrast study axial section, the bilobed mass lesion is noted in the carotid sheath on the right side of the neck at the bifurcation of the right CCA causing splaying of the ICA and ECA called Lyre´s sign. It shows intense homogeneous enhancement. Multiple thin tortuous channels are noted around this lesion (Figure 5, Figure 6). According to the Shamblin group system, the tumor's circumferential angle of contact with ICA is a group I (<180 degrees of encasement).

**Diagnosis:** a diagnosis of the well-defined homogeneously enhancing lesion at the bifurcation of the right CCA suggestive of carotid body tumor was made.

**Therapeutic interventions:** the patient underwent surgical resection of a neck tumor. Post-operative image showing bilobed mass lesion. On histopathological examination, the definitive diagnosis came as carotid body paraganglioma.

**Follow-up and outcome of interventions:** the patient returned to the clinic for follow-up but the further visits were inconclusive.

**Patient perspective:** a patient was happy after receiving successful treatment.

**Informed consent:** informed written consent was given by the patient.

## Discussion

A paraganglioma is a tumor that can develop anywhere in the body from paraganglia, which are non-neuronal cells descended from neural crest cells [5]. The carotid body, the vagus nerve in the head and neck region, the jugular foramen, the middle ear, the aortopulmonary window, and the organ of Zuckerkandl are common sites. The manifestations of carotid body paraganglioma might be sporadic or familial. The sporadic type has no preference for gender and is typically encountered in people between the ages of 45 and 50. Its cause is unknown. A lateral neck lump with modest growth and no pain is how lesions appear. Cranial nerve palsies, voice changes, and hearing loss are some of the accompanying symptoms [6,7]. The carotid body is located in the carotid sheath. It is a round, reddish-brown organ that is highly specialized, well-circumscribed, and situated in the adventitia of the carotid bifurcation. It receives nourishment from feeding vessels, which are largely supplied by the ascending pharyngeal branch of the ECA. The nerve supply of the tumor is by the vagus nerve and glossopharyngeal nerve. The carotid body typically has a diameter of 2 to 6 mm, but those who live at higher elevations frequently have bigger carotid bodies. Acidosis, hypoxia, and hypercapnia trigger the chemoreceptor organ that regulates blood pressure, heart rate, respiration, and blood temperature by increasing sympathetic flow [8].

The majority of the time, CBTs are discovered accidentally on imaging studies or through clinical assessment. Image analysis and pathological anatomy play a major role in the final diagnosis. Doppler ultrasound, CECT neck, contrast enhance magnetic resonance imaging (MRI) neck, and angiography plays an important role in the diagnosis of CBT. The best screening method for CBTs is a color-flow carotid duplex, and CBT is characterized by a well-defined hypoechoic mass causing splaying of the ICA and ECA. On color Doppler imaging hypervascularity is seen within the lesion with a low-resistance flow pattern. The “gold standard” diagnostic method for CBTs in the past was angiography, which provided a precise delineation of the vascular supply and confirmed the diagnosis. Surgical tumor resection depends on the relationship of the tumor with the artery bifurcation and the proximity of cranial nerves diagnosed accurately on CT angiography (CTA) or MR angiography (MRA). The accuracy of MRI is frequently higher than that of CT because it does not involve ionizing radiation. CTA can characterize the tumor's size and relationship to bony landmarks, which can change the surgical strategy. Other head/neck paragangliomas and contralateral tumors are also plainly discernible. On dynamic contrast-enhanced MRI and diffusion-weighted imaging (DWI), paragangliomas are better differentiated from other benign neoplastic tumors. On digital subtraction angiography (DSA), a tumor with an “early vein” visible due to arteriovenous shunting is detected by a high blush and the splaying of the carotid vessels (lyre sign).

The primary contributing supply is the ascending pharyngeal artery. Successful diagnosis can be made using ultrasound and CT. An MRI was obtained for preoperative assessment. The left ECA and ICA were splayed as a result of a well-defined hypoechoic mass at the CCA bifurcation that had enhanced vascularity. On CT, this lesion was clearly enhanced and showed a soft tissue density. On MRI, the mass appeared hypointense on T1 weighted imaging (WI) and hyperintense on T2WI. Diffusion-weighted imaging showed a true restriction corresponding to low apparent diffusion coefficient (ADC) values. Our case is similar to the characteristics of the research of Ying Yuan *et al*. [9]. That is CBTs demonstrate well-circumscribed and intensely enhancing masses.

In our case, a thorough evaluation of a CBT, including history, clinical examination, and investigations, was carried out in order to reach a provisional diagnosis. The purpose of radiography is to identify CBTs and their effects, but surgery is the only way to make a definitive histological diagnosis.

## Conclusion

Carotid body tumors are rare, hypervascular, slow-growing neuroendocrine tumors. A preliminary diagnosis can be made on the patient's medical history, physical examination, and radiography. They frequently splay the ICA and ECA when imaging. Surgical resection is the preferred course of treatment for CBTs.
